# Koalas in continuous time – unravelling the movement ecology of an iconic species using insights from a statewide telemetry program

**DOI:** 10.1186/s40462-026-00693-3

**Published:** 2026-08-12

**Authors:** M. Le Pla, L. Vogelnest, T. Jessop

**Affiliations:** 1 Koala Science Team, Conservation and Restoration Science, Science and Insights Division, Department of Climate Change, Energy, The Environment and Water (DCCEEW), Locked Bag 5022, Parramatta, New South Wales 2124 Australia; 2 Taronga Conservation Society Australia, Sydney, Australia

## Abstract

Movement ecology has entered an era of unprecedented data abundance, yet contemporary GPS datasets present analytical challenges due to temporal autocorrelation between consecutive locations. Continuous-time movement modelling (CTMM) addresses these challenges by treating movement as a stochastic process unfolding continuously through space and time, producing parameter estimates of movement metrics that are invariant to sampling schedule. Here we apply CTMM to a comprehensive GPS telemetry dataset from 230 koalas (*Phascolarctos cinereus*) tracked across seven populations throughout New South Wales, Australia – a first for this species at scale. Our primary aim was to characterise fundamental movement attributes and provide evidence-based recommendations for telemetry study design in this threatened species. All koala datasets exhibited clear evidence of autocorrelation, and we observed substantial variation in home range size and diffusion rates among study regions, consistent with broad environmental gradients spanning productive coastal and riparian habitats to drier, fragmented inland landscapes. Despite 7–10 fold variation in home range size and diffusion rates at a population level, the temporal structure of koala movement was remarkably consistent across all populations. Range crossing times and directional persistence timescales showed little regional variation, suggesting these timescales reflect intrinsic biological constraints rather than being driven by local environmental conditions. Sexual dimorphism in space use and movement rates was also consistent across all regions: male home ranges were approximately twice the size of females regardless of local habitat conditions, and males consistently moved throughout broader areas each day. These autocorrelation timescales have important implications for telemetry study design and broader monitoring programs. Irrespective of sampling frequency, the slow range crossing time of koalas indicates that deployments of at least 3–5 weeks are necessary for reliable home range estimation. Directional persistence timescales suggest fix intervals under three hours are required to resolve fine-scale movement behaviors such as speed and distance travelled. When identifying contact events between individuals is an objective, high resolution sampling (i.e. 30 min or less) is required. This study demonstrates how CTMM can extract fundamental movement parameters from GPS data to simultaneously address autocorrelation in movement trajectories, advance ecological understanding and optimize future monitoring efforts for threatened species.

**Supplementary Information:**

The online version contains supplementary material available at https://doi.org/10.1186/s40462-026-00693-3.

## Clinical trial number

Not applicable.

**Supplementary Information:**

The online version contains supplementary material available at https://doi.org/10.1186/s40462-026-00693-3.

## Introduction

Movement ecology has entered an era of unprecedented data abundance [1, 2]. Technological advances in GPS telemetry now enable researchers to collect precise location data at fine temporal resolutions over extended periods, generating datasets orders of magnitude larger than those available just a decade ago [2, 3]. The scope of contemporary GPS datasets is compelling: more frequent observations should, at face value, provide richer insights into animal space use, movement patterns, and the ecological processes that shape them. However, the relationship between data quantity and ecological information is not straightforward. As fix intervals decrease and sample sizes grow, the statistical assumptions underlying traditional analytical approaches become increasingly violated, particularly regarding temporal autocorrelation [4–6]. Consecutive GPS locations are inherently non-independent, with the degree of autocorrelation intensifying as animals are observed more frequently. This presents a fundamental paradox: contemporary GPS technology allows one to collect more data than ever before, yet more data does not necessarily translate to better inference when analyzed using methods that fail to account for the temporal structure inherent in frequently sampled movement trajectories (e.g., [7, 8]).

Continuous-time movement modelling (CTMM) offers a framework to address these analytical challenges [5, 9–11]. CTMM approaches model movement as a stochastic process unfolding continuously through space and time, with GPS fixes representing imperfect samples from this underlying process. This paradigm shift brings several fundamental advantages. First, CTMM produces parameter estimates that are invariant to sampling schedule, enabling direct comparisons between datasets collected at different temporal resolutions or with different GPS technologies [4, 8]. Second, when calibration data are available, CTMM can explicitly incorporate location error attributable to the observation process, propagating measurement uncertainty through all subsequent analyses and producing more realistic confidence intervals on derived parameters such as home range size and movement rates [12]. Third, CTMM employs sophisticated likelihood-based approaches that appropriately weight observations according to their autocorrelation structure, extracting maximal information from densely sampled trajectories without the statistical violations inherent in treating autocorrelated observations as independent data points [13, 14]. Explicitly accounting for autocorrelation in movement datasets has clear inferential benefits: traditional kernel density estimators applied to autocorrelated movement data often systematically underestimate home range size [8], and assuming statistical independence of locations can lead to both pseudo-replication and bias in resource selection function parameter estimates [7].

The analytical power of CTMM becomes particularly valuable when applied to datasets spanning broad environmental gradients, scales of movement and sampling regimes (e.g., [8]). By fitting CTMM models to GPS data collected simultaneously across multiple populations experiencing different ecological conditions, researchers can estimate fundamental movement parameters – including range crossing times, directional persistence timescales, diffusion rates, and asymptotic home range sizes – in a statistically robust framework that facilitates direct comparisons. Understanding the timescales over which distinct aspects of the movement process unfold is not just useful from an ecological perspective, it also allows researchers to design sampling protocols that maximize information gain while minimizing costs, animal welfare concerns, and downstream analytical complications [14, 15].

Koalas (*Phascolarctos cinereus*) are an ideal study species for demonstrating the advantages of CTMM approaches to modelling animal movements. As arboreal folivores with relatively sedentary lifestyles, koalas move between trees in discrete bouts before settling into extended periods of feeding or rest [16, 17]. Koala populations face multiple interacting threats including habitat loss and fragmentation [18, 19], vehicle strikes [20, 21], dog attacks [22], disease [23, 24], and climate change [25, 26]. Consequently, the effectiveness of management interventions – including corridor design, road crossing mitigation, translocation programs and habitat protection – all fundamentally depend on a robust understanding of how koalas move through the landscape. Whilst a small number of recent studies have adopted CTMM approaches to estimate koala home ranges (e.g., [27–29]), there has been little attention paid to the broader suite of movement parameters estimated by fitting CTMM models during home range estimation procedures. These parameters – particularly range crossing time and directional persistence timescales – represent fundamental descriptors of movement behavior that remain uncharacterised for koalas and yet are essential for both understanding their ecology and designing optimal telemetry or population monitoring programs (e.g. size of survey area and timing of repeat surveys). Moreover, no previous study has leveraged CTMM-derived parameter estimates to provide explicit guidance on telemetry study design for koalas, despite the considerable logistical and financial costs of GPS collar deployments and the ethical imperative to maximize information gained from each deployed device ([30, 31]).

Here we present the first comprehensive assessment of koala movement behavior across a broad gradient of environmental conditions and at a population scale by applying CTMM to a GPS telemetry dataset comprised of 230 koalas tracked across seven populations over three years (2023–2026) as part of the NSW Sentinel Koala Population Monitoring Program. This program has gained detailed baseline information, including reproduction, mortality, dispersal, genomic diversity, population connectivity, inbreeding, diet, microbiome, disease prevalence and movement behavior across seven key locations, or sentinel sites, for NSW koalas. This program is led by the NSW Government (Department of Climate Change, Energy, Environment, and Water), in collaboration with Taronga Conservation Society Australia, The University of Sydney, The Australian National University, and the University of Western Sydney. This dataset provides an unprecedented opportunity to estimate population-level movement parameters while accounting for individual heterogeneity, measurement error, and temporal autocorrelation. This study constitutes the first application of CTMM to koalas at scale and adds to a small pool of studies aiming to characterise variation in autocorrelation timescales among different populations of the same species living in different habitat contexts (e.g., [32, 33]). We then describe how these fundamental aspects of koala movement ecology support evidence-based recommendations for the design of GPS telemetry studies in this and other species with similar movement characteristics.

## Methods

### Study areas

**Fig. 1:**
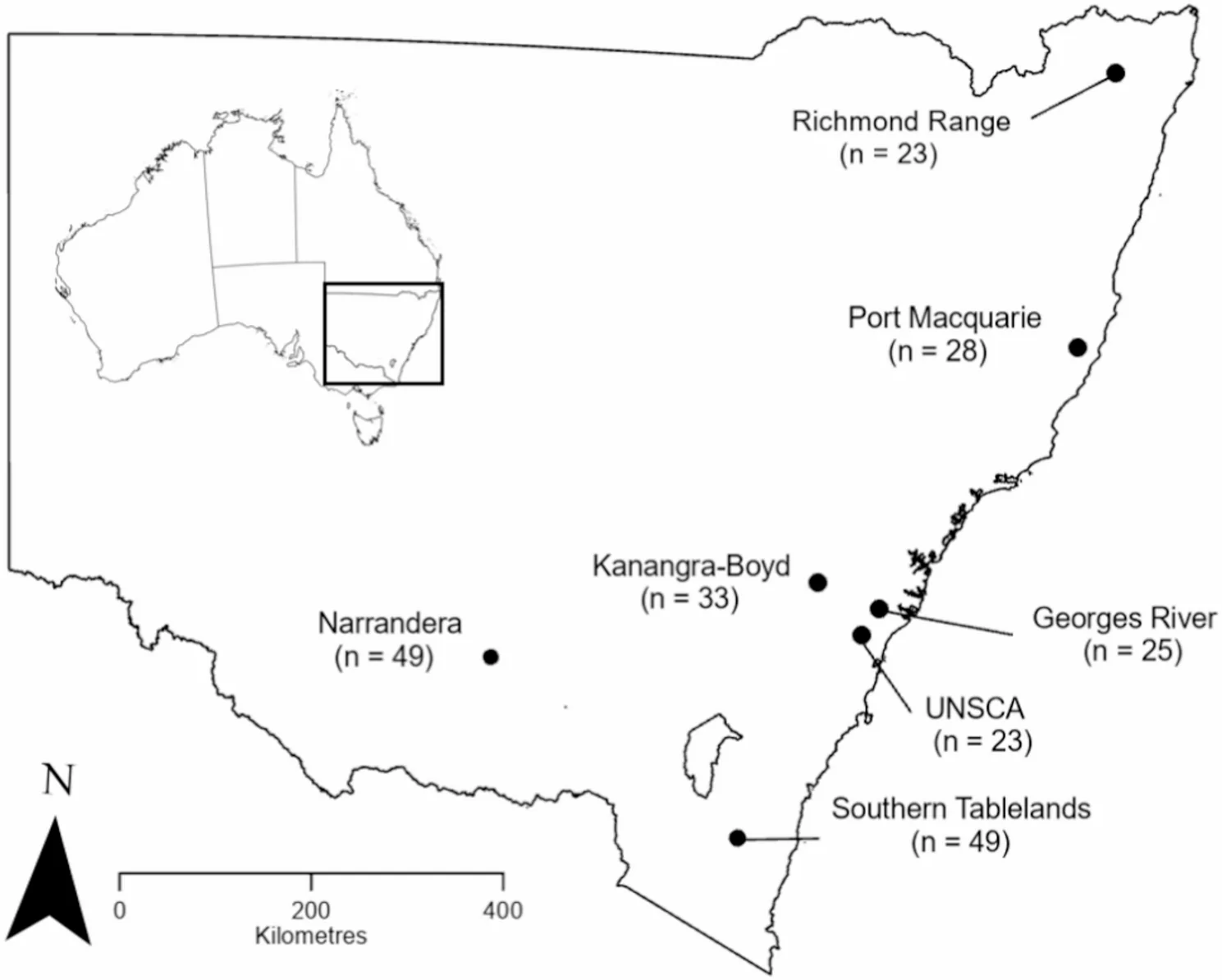
Sentinel study locations and respective sample sizes of GPS collared koalas used in this study

Study sites for the Sentinel program are located in the Port Macquarie Conservation Areas (hereafter Port Macquarie; 31.435° S, 152.900° E), Richmond Range National Park (hereafter Richmond Range; 28.807°S, 152.742°E), the Narrandera Common and Conservation Areas (hereafter Narrandera; 34.748° S, 146.550° E), Kanangra-Boyd National Park (hereafter Kanangra-Boyd; 33.832° S, 149.999° E), Georges River Koala National Park (hereafter Georges River; 34.111° S, 150.810° E), the Upper Nepean State Conservation Area (hereafter UNSCA; 34.443° S, 150.523° E), and the Southern Tablelands Conservation Areas (hereafter Southern Tablelands; 34.885° S, 149.472° E) (Fig. 1). These sites were chosen to be geographically and climatically representative of koala distributions in NSW, as perceived important koala strongholds with large populations, logistically accessible for field teams, a value-add for building on previous research, and considering local stakeholders including First Nations communities and the cultural significance of koalas in those areas.

### Koala tracking data collection

Koalas were sampled during routine Koala Sentinel population monitoring between 2023 and 2026. Sites were sampled once per year. Koalas were located via thermal drone and captured using the ‘guide rope and flag’ method [34, 35]. Individuals were anaesthetised with alfaxalone (2–3 mg/kg) and medetomidine (0.05 mg/kg) intramuscularly, with anaesthesia supplemented as needed using alfaxalone (0.8–1 mg/kg) or isoflurane. Medetomidine was reversed with atipamezole (5× the medetomidine dose). A range of samples and morphometric data were collected. GPS collars (K-tracker units; https://www.lx-group.com.au/portfolio/k-tracker-animal-tracking-devices) were fitted to a subset of captured koalas that were sexually mature (females aged 3-3.5 years; males 4-4.5 years) and large enough (based on body size and weight) that they would not outgrow the collar before scheduled recapture the following year.

Collar deployments encompassed a variety of fix-frequency schedules to serve multiple research objectives, including 12-hourly, 6-hourly, 2-hourly and half-hourly intervals. Additionally, K-tracker GPS collars also include a feature to record additional locations when collars from different individuals were close to each other, which happened intermittently throughout collar deployments. Telemetry datasets with irregular fix intervals such as these present significant challenges to movement analyses conducted using discrete-time analytical methods as they often require the analyst to discard substantial data to ensure regularity in trajectories [4, 5]. However, regularization of movement trajectories to a consistent sampling interval was not required for the purposes of our study as irregular fix frequency is explicitly accounted for in analyses grounded in CTMM frameworks.

### Data filtering, preparation and error calibration

To characterise baseline movement behavior representative of routine space use patterns in koalas, we excluded GPS data associated with koala breeding activity. In NSW, koala breeding typically occurs from August to February, with increased movement often peaking between September and December [36, 37]. Consequently, we focused our analyses on range-resident koalas during periods when movements were unlikely influenced by breeding-related activities by first filtering GPS data to that collected between the 1st of February and 31st of July each year.

GPS location error was then characterised for each population using calibration data collected from K-tracker collars that had their weak-link broken during deployments. We used data from these stationary collars (*n* = 97 collars, range: 5–23 collars per region, 32633 fixes) to estimate the User Equivalent Range Error (UERE) for each device and population combination using Position Dilution of Precision (PDOP) values (Table S1; [12]). Because these population-specific error models scale the calibrated UERE by the PDOP value recorded at each fix, estimated location error varies among individual fixes (Fig. S1). The resulting region-specific error models, which account for both GPS device error and local environmental factors affecting satellite communication quality (e.g. canopy cover), were then incorporated into all subsequent movement model fitting and home range estimation procedures. This calibration step ensured that location uncertainty was quantified and propagated through to movement parameter estimates and their associated confidence intervals [12]. We then assessed each koala’s dataset for location and speed outliers by using the outlie function within *ctmm* package. Locations were considered outliers if they were far from the median location and if the minimum speed required to explain the displacement was implausible for koalas.

Individual trajectories were then visually assessed to identify and remove any clear short-term extraterritorial forays [38]. Extraterritorial forays were defined as movements that were clearly beyond the area an individual occurred for most of their tracking duration, directionally persistent in nature, maintained for several days and directed to areas that were either never again visited during collar deployments, or only revisited during peak breeding season (September – October). Where there was limited data in the non-breeding season alone to determine if movements were typical or extraterritorial forays, we compared potential forays to overall space use patterns throughout their entire tracking period. Forays were removed from koala datasets to meet the assumption of statistical stationarity in movement behavior prior to movement model fitting, and to ensure any predicted home range areas and movement parameter estimates reflected the typical movement behavior of range resident koalas (Fig. S2).

Some individuals were tracked for more than one year or recaptured and recollared during the second year of the Sentinel program if their collars had dropped. In these cases, telemetry data was combined across non-breeding seasons (2024, 2025 and 2026) and visually assessed to determine if range shifts occurred between years. There was no clear evidence of range shifts between years, with fixes from different non-breeding seasons largely occurring within the same general area as prior years.

### Continuous-time movement modelling

After pre-processing, we then used variogram analysis to determine if koalas were range-resident or transient [4]. Individuals were considered range-resident when a clear plateauing of semivariance was evident at increasing time lags and transient when semivariance continued to increase over time (Fig. S3; [4]).

Prior to movement model fitting, we generated an initial parameter guess with anisotropic (elliptical covariance) movement and telemetry error explicitly enabled. We then fitted and selected an autocorrelated movement model that best described each koala’s movements using perturbative Hybrid Residual Maximum Likelihood (phREML; [39]) and Akaike’s Information Criterion corrected for small sample sizes (AIC_C_) [40]. Several movement models are considered in this approach including an IID (independent and identically distributed) movement process that has no temporal autocorrelation; an OU (“Ornstein-Uhlenbeck”) movement process that features autocorrelation in an animal’s position but no autocorrelation in velocity [41]; and an OUF (“Ornstein-Uhlenbeck Foraging”) movement process that features autocorrelation in both position and velocity [4, 5].

Fitted autocorrelated movement models are characterised by several key parameters that describe different aspects of the movement process. These parameters include the position autocorrelation timescale, Ƭ_p_, the timescale over which autocorrelation in spatial locations decays to statistical independence (i.e. how long it takes an animal to cross its home range ~ *range crossing time*, [39]); the velocity autocorrelation timescale, Ƭ_v_, the timescale over which autocorrelation in velocity decays to statistical independence (i.e. how long an animal persists in its motion ~ *directional persistence*, [32]); and the diffusion rate (D), the expected square displacement over a finite period of time (i.e. the variance in locations per unit of time, [42]).

Home ranges of range-resident koalas were then estimated using weighted autocorrelated kernel density estimation (wAKDE; [14, 43]). wAKDE weights observations according to their autocorrelation structure as described by the selected movement models [14, 43], producing unbiased home range estimates that properly account for temporal autocorrelation in movement data. We present 95% isopleth home range estimates accompanied by 95% confidence intervals.

### Population-level parameter estimation

To characterise population-level patterns in movement behavior and quantify differences between sexes and study regions, we conducted meta-analyses of individual movement parameters [44]. This hierarchical approach accounts for individual heterogeneity in parameter estimates and their associated uncertainty, producing population-level mean estimates and confidence intervals that properly incorporate both within-individual uncertainty and among individual variation [44]. Individual estimates of home range size, Ƭ_p,_ Ƭ_v_, and D are treated as having a 𝜒^2^ sampling distribution, and population mean estimates as having an inverse-Gaussian (*IG*) distribution [44, 45]. To ensure individual parameter estimates used are broadly representative of range-resident koala movement behavior, individuals with survey durations less than 20 days were excluded from population-level parameter estimation procedures.

In addition to population mean estimates, the meta-analysis framework provides estimates of the coefficient of variation (CoV), which quantifies heterogeneity in movement parameter estimates within a population after accounting for estimation uncertainty [44]. A CoV of 1 indicates that the standard deviation among individuals equals the population mean, representing intermediate heterogeneity. Values closer to 0 indicate less heterogeneity among individual estimates and values substantially greater than 1 indicate high heterogeneity among individual estimates.

To quantify differences in movement parameters within and between regions and sexes, we estimated effect sizes as ratios of population means [44]. Ratio-based effect sizes provide more informative comparisons than p-values alone, as they directly quantify the magnitude of differences between groups while communicating uncertainty through confidence intervals [44, 46]. A ratio confidence interval that contains 1 indicates a non-significant difference but may still contain ecologically substantial ratios (e.g. 1.5 or 2), indicating that a meaningful difference cannot be ruled out. Conversely, a confidence interval that excludes 1 but remains close to it (e.g. 1.01–1.05) indicates a significant but potentially trivial difference. This framework avoids conflating statistical significance with biological importance [44].

We conducted two sets of comparisons. First, to characterise the regional gradient in space use and movement behaviour, we compared the population mean home range area, diffusion rate, range crossing time and directional persistence timescale of koalas from the region with the smallest population mean estimate (the reference population) to other regions separately for males and females respectively. Second, to quantify intra-region sexual dimorphism in movement parameters, we compared females (the reference population) to males within each region.

### Data analysis and software

All analyses were conducted in the R programming environment (version 4.4.1; [47]). Error calibration, outlier removal, movement model fitting and home range estimation was conducted using the *ctmm* package (version 1.3.1; [48]).

## Results

A total of 230 koalas (80 males and 150 females) had sufficient data in the non-breeding season for continuous-time movement model fitting after pre-processing and filtering, resulting in a dataset comprised of 302,954 GPS locations. Sample sizes varied between monitoring regions, ranging from 23 koalas in Richmond Range and UNSCA to 49 koalas in Narrandera and the Southern Tablelands (Table 1; Fig. 1). Individual collar monitoring durations varied widely within and between regions, ranging from 22 days to 358 days (i.e. two full non-breeding seasons). Mean survey duration of GPS-collared koalas across the entire dataset was 140 days and ranged from 90 days in Narrandera to 174 days in Georges River (Table 1). Extra-territorial forays made up a small proportion of the total fixes collected both overall (4.3%) and within each region (range: 0.3% – 8.7%) (Table S2).

**Table 1:** Summary of GPS tracking data collected from koalas during the non-breeding season (February-July) across seven monitoring sites in New South Wales, 2024–2026

Region	Males	Females	Days	Fixes
Port Macquarie	9	19	107 (22–266)	51,844
Narrandera	18	31	90 (27–225)	26,242
Richmond Range	9	14	136 (48–276)	75,163
Kanangra-Boyd	11	22	170 (46–358)	29,945
Georges River	6	19	174 (48–352)	36,241
UNSCA	10	13	150 (49–334)	36,425
Southern Tablelands	17	32	156 (32–311)	47,094
**Total**	**80**	**150**	**140 (22–358)**	**302,954**

Days represent the mean tracking duration of koalas per region (range in parentheses). Fixes represent the total number of GPS locations recorded per region.

### Range residency

Visual assessment of variograms indicated the majority of koalas in this study were range resident (*n* = 212, ~ 92%). However, except for Kanangra-Boyd and Narrandera, all other regions also had koalas that were considered transient during the study period (*n* = 19, ~ 8%, Table 2). Transient koalas made up a small proportion of GPS-collared animals in the Southern Tablelands, Georges River, Port Macquarie and UNSCA regions (6%, 8%, 11% and 13% respectively), and a larger proportion of GPS collared animals in Richmond Range (30%) (Table 2). Overall, a similar proportion of males and females were considered transient (9% and 7% of GPS collared animals respectively).

Of the 212 range-resident koalas, 159 (~ 75%) were best characterised by the OUF movement process and 53 (~ 25%) were best characterised by the OU movement process (Table S3). Except for Kanangra-Boyd, the OUF movement process was supported for the majority of koalas within each region (range: 69% – 93%, Table S3). The IID movement process was not supported for any koala movement dataset.

**Table 2:** Sample sizes of male and female koalas deemed range resident or transient during the study period within each region

Region	Range Residents		Transients	
	Male	Female	Male	Female
Port Macquarie	8	17	1	2
Narrandera	18	31	0	0
Richmond Range	6	10	3	4
Kanangra-Boyd	11	22	0	0
Georges River	6	17	0	2
UNSCA	9	11	1	2
Southern Tablelands	15	31	2	1
**Total**	**73**	**139**	**7**	**11**

Animals are deemed range resident when a clear plateauing of semivariance was evident at increasing time lags and transient when semivariance continued to increase over time.

### Home range size

**Fig. 2:**
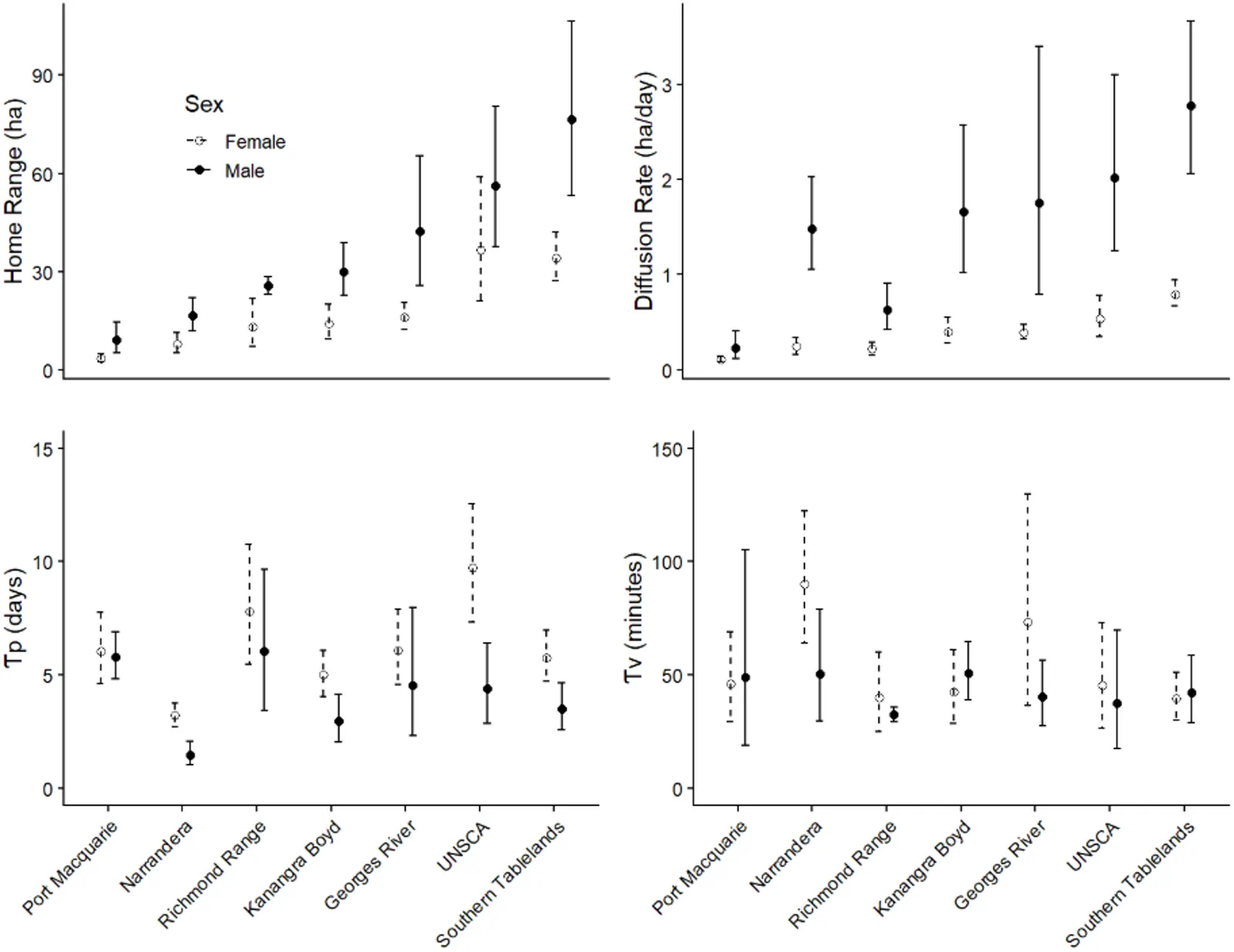
Population-level movement parameter estimates derived from continuous-time movement models for male (solid points and lines) and female (hollow points and dashed lines) koalas across seven monitoring sites in New South Wales. Parameters include 95% home range area (ha) (top left), diffusion rate (ha/day) (top right), range crossing time (Ƭ_p_, days) (bottom left), and directional persistence timescale (Ƭ_v_, minutes) (bottom right). Points represent meta-analytic mean estimates with 95% confidence intervals. Sites are ordered by increasing home range size

Koala home range sizes varied widely within and between regions, following a broad gradient of small (Port Macquarie and Narrandera) through to large (UNSCA and Southern Tablelands) (Fig. 2). The smallest range-resident home range was 0.64 ha (95% CI: 0.39–0.94 ha, female, Port Macquarie) and the largest range-resident home range was 179.39 ha (95% CI: 111.30–263.46 ha, male, Southern Tablelands). Mean effective sample sizes for home range estimation varied among regions (range: 16–54) and individuals (range: 3–347), largely reflecting variation in survey duration and range crossing times among GPS tracked koalas (Table S4).

Population-level male home range estimates were 9.19 ha (95% CI: 5.31–14.75 ha) in Port Macquarie; 16.57 ha (95% CI: 12.06–22.17 ha) in Narrandera; 25.74 ha (95% CI: 23.03–28.59 ha) in Richmond Range; 30.05 ha (95% CI: 22.73–38.91 ha) in Kanangra-Boyd; 42.44 ha (95% CI: 25.78–65.34 ha) in Georges River; 56.28 ha (95% CI: 37.69–80.42 ha) in UNSCA; and 76.56 ha (95% CI: 53.30–106.29 ha) in Southern Tablelands (Fig. 2).

Female home range sizes followed the same regional trend, with population-level female home range estimates of 3.64 ha (95% CI: 2.49–5.11 ha) in Port Macquarie; 7.90 ha (95% CI: 5.24–11.37 ha) in Narrandera; 13.08 ha (95% CI: 7.17–21.85 ha) in Richmond Range; 14.08 ha (95% CI: 9.45–20.16 ha) in Kanangra-Boyd; 16.18 ha (95% CI: 12.39–20.71 ha) in Georges River; 36.59 ha (95% CI: 20.00–58.93 ha) in UNSCA; and 34.11 ha (95% CI: 27.26–42.09 ha) in Southern Tablelands (Fig. 2).

### Diffusion rate

Diffusion rate estimates revealed koalas typically moved throughout small areas within a 24-hour period in all regions, with regional-level variation in daily movement rates broadly aligning with trends in home range size (Fig. 2). The smallest diffusion rate was 0.03 ha/day (95% CI: 0.02–0.04 ha/day, female, Port Macquarie) and the largest diffusion rate was 5.50 ha/day (95% CI: 4.97–6.05 ha/day, male, Southern Tablelands). Effective sample sizes for diffusion rate estimation varied among regions (mean range: 215–545) and individuals (range: 15–1772) (Table S4).

Population-level male diffusion rates were 0.23 ha/day (95% CI: 0.12–0.41 ha/day) in Port Macquarie; 1.48 ha/day (1.05–2.03 ha/day) in Narrandera; 0.63 ha/day (95% CI: 0.42–0.91 ha/day) in Richmond Range; 1.66 ha/day (95% CI: 1.02–2.57 ha/day) in Kanangra-Boyd; 1.75 ha/day (95% CI: 0.79–3.40 ha/day) in Georges River; 2.02 ha/day (95% CI: 1.25–3.10 ha/day) in UNSCA; and 2.86 ha/day (95% CI: 2.15–3.74 ha/day) in Southern Tablelands (Fig. 2).

Female diffusion rates were typically smaller than males in all regions (Fig. 2). Population-level, female diffusion rates were 0.11 ha/day (95% CI: 0.08–0.14 ha/day) in Port Macquarie; 0.24 ha/day (0.16–0.34 ha/day) in Narrandera; 0.22 ha/day (95% CI: 0.15–0.29 ha/day) in Richmond Range; 0.40 ha/day (95% CI: 0.28–0.55 ha/day) in Kanangra-Boyd; 0.39 ha/day (95% CI: 0.32–0.47 ha/day) in Georges River; 0.53 ha/day (95% CI: 0.35–0.78 ha/day) in UNSCA; and 0.79 ha/day (95% CI: 0.67–0.94 ha/day) in Southern Tablelands (Fig. 2).

### Range crossing time

There was little variation in male range crossing times (Ƭ_p_) across the study regions (Fig. 2). Population-level male range crossing times were 5.81 days (95% CI: 4.82–6.90 days) in Port Macquarie; 1.49 days (95% CI: 1.04–2.07 days) in Narrandera; 6.05 days (95% CI: 3.45–9.66 days) in Richmond Range; 2.98 days (95% CI: 2.06–4.17 days) in Kanangra-Boyd; 4.54 days (95% CI: 2.32–7.98 days) in Georges River; 4.40 days (95% CI: 2.87–6.40 days) in UNSCA; and 3.52 days (95% CI: 2.58–4.67 days) in Southern Tablelands (Fig. 2). Except for Narrandera, male range crossing time estimates for each region had broadly overlapping 95% confidence intervals with several other regions (Fig. 2).

Similarly, whilst female range crossing times were typically longer than males, there was little variation among regions (Fig. 2). Population-level female range crossing times were 6.05 days (95% CI: 4.62–7.76 days) in Port Macquarie; 3.23 days (95% CI: 2.74–3.76 days) in Narrandera; 7.81 days (95% CI: 5.46–10.75 days) in Richmond Range; 5.00 days (95% CI: 4.05–6.08 days) in Kanangra-Boyd; 6.09 days (95% CI: 4.59–7.91 days) in Georges River; 9.73 days (95% CI: 7.35–12.54 days) in UNSCA; and 5.78 days (95% CI: 4.74–6.97 days) in Southern Tablelands (Fig. 2). Except for Narrandera, most female range crossing times for each region had broadly overlapping 95% confidence intervals with several other regions (Fig. 2). Mean range crossing times across all regions was 3.67 days (95% CI: 2.95–4.50) for males and 5.69 days (95% CI: 5.13–6.30) for females.

### Directional persistence

Male and female directional persistence time scales (Ƭ_v_) were similar across our study regions (Fig. 2). Population-level male directional persistence time scales were 49.20 min (95% CI: 19.20–105.00 min) in Port Macquarie; 50.40 min (95% CI: 29.76–79.20 min) in Narrandera; 40.20 min (95% CI: 25.20–60.00 min) in Richmond Range; 51.00 min (95% CI: 39.00–64.80 min) in Kanangra-Boyd; 40.47 min (95% CI: 27.73–56.56 min) in Georges River; 37.56 min (95% CI: 17.46–69.66 min) in UNSCA; and 42.10 min (95% CI: 29.04–58.86 min) in Southern Tablelands (Fig. 2).

Population-level female directional persistence time scales were 46.20 min (95% CI: 29.40–69.00 min) in Port Macquarie; 90.18 min (95% CI: 64.20–122.46 min) in Narrandera; 49.80 min (95% CI: 25.20–86.40 min) in Richmond Range; 42.60 min (95% CI: 28.80–61.20 min) in Kanangra-Boyd; 73.20 min (95% CI: 36.60–129.72 min) in Georges River; 45.48 min (95% CI: 26.46–72.96 min) in UNSCA; and 39.62 min (95% CI: 30.01–51.23 min) in Southern Tablelands (Fig. 2). Mean directional persistence timescale across all regions was 42.48 min (95% CI: 35.14–50.82) for males and 55.74 min (95% CI: 47.04–65.64) for females.

### Individual heterogeneity and effect size ratios

Individual heterogeneity (CoV) in male and female home range sizes within each region was moderate; ranging from 0.44 (95% CI: 0.23–0.66) in Kanangra-Boyd to 0.73 (0.25–1.21) in Port Macquarie for males, and from 0.59 (95% CI: 0.42–0.77) in Southern Tablelands to 1.03 (0.66–1.38) in Narrandera for females (Table S5). Using Port Macquarie as the reference population, effect size ratios revealed 8-fold variation in male home range sizes and 9-fold variation in female home range sizes among our study regions (Table 3).

Similarly, individual heterogeneity in male and female diffusion rates within each region was moderate; ranging from 0.51 (95% CI: 0.21–0.82) in Richmond Range to 0.99 (95% CI: 0.35–1.66) in Georges River for males, and from 0.41 (95% CI: 0.27–0.55) in Georges River to 1.05 (95% CI: 0.71–1.38) in Narrandera for females (Table S5). Using Port Macquarie as the reference population, effect size ratios revealed 10-fold variation in male diffusion rates and 7-fold variation in female diffusion rates among our study regions (Table 3).

Individual heterogeneity in male and female range crossing times within each region was low to moderate; ranging from 0.54 (95% CI: 0.30–0.77) in the Southern Tablelands to 0.78 (95% CI: 0.27–1.30) in Georges River for males, and from 0.35 (95% CI: 0.15–0.56) in UNSCA to 0.53 (95% CI: 0.31–0.75) in Georges River for females (Table S5). Using Port Macquarie as the reference population, effect size ratios revealed male koalas crossed their range 4-fold faster in Narrandera, 2-fold faster in Kanangra-Boyd, UNSCA and the Southern Tablelands regions, and at similar timescales in Richmond Range and Georges River (Table 3). In contrast, most female range crossing times, with the exception of Narrandera and UNSCA, had confidence intervals overlapping 1 indicating no statistically detectable differences in range crossing time among regions (Table 3). Relative to Port Macquarie females, Narrandera females crossed their range twice as fast and UNSCA females took 50% longer to cross their range (Table 3).

Individual heterogeneity in male and female directional persistence timescales was moderate; ranging from 0.39 (95% CI: 0.12–0.67) in Georges River to 1.20 (95% CI: 0.36–2.07) in Port Macquarie for males, and from 0.56 (0.24–0.88) in Kanangra-Boyd to 1.09 (0.50–1.68) in Georges River for females (Table S5). Due to small sample sizes of animals whose velocity autocorrelation could be adequately resolved (i.e. OUF models selected), CoV for directional persistence timescales could not be estimated in males for the Richmond Range and Kanangra-Boyd regions. Using Port Macquarie as the reference population, effect size ratios revealed no statistically detectable differences in directional persistence timescales among regions for males (Table 3). Similarly, there were no differences in directional persistence timescales among most regions for females except for Narrandera where females maintained directional motion for 85% longer than the reference population (Port Macquarie) (Table 3).

**Table 3:** Between region effect size ratios for male – male and female - female home range areas, diffusion rates, range crossing times and directional persistence timescales using Port Macquarie as the reference population (ratio = 1)

Region	Home Range Size (95% CI)		Diffusion Rate (95% CI)	
	Male	Female	Male	Female
Port Macquarie	1	1	1	1
Narrandera	1.68 (0.85–3.11)	2.10 (1.17–3.48)	5.71 (2.55–11.94)	2.19 (1.29–3.48)
Richmond Range	2.60 (1.45–4.61)	3.47 (1.60–6.37)	2.42 (1.04–5.16)	1.97 (1.21–3.06)
Kanangra-Boyd	3.04 (1.58–5.58)	3.74 (2.09–6.17)	6.42 (2.59–14.02)	3.65 (2.20–5.70)
Georges River	4.35 (1.98–8.53)	4.18 (2.54–6.62)	6.76 (2.01–16.57)	3.65 (2.43–5.37)
UNSCA	5.59 (2.29–11.53)	9.44 (4.50–17.15)	7.81 (3.17–16.98)	5.02 (2.78–8.26)
Southern Tablelands	7.94 (3.87–15.07)	9.34 (5.93–14.30)	10.73 (4.91–22.14)	7.29 (4.98–10.50)
	**Range Crossing Time (95% CI)**		**Directional Persistence (95% CI)**	
	**Male**	**Female**	**Male**	**Female**
Port Macquarie	1	1	1	1
Narrandera	0.26 (0.17–0.37)	0.52 (0.38–0.71)	0.81 (0.24–2.33)	1.85 (1.03–3.15)
Richmond Range	1.05 (0.56–1.70)	1.27 (0.80–1.90)	0.52 (0.18–1.42)	0.83 (0.42–1.45)
Kanangra-Boyd	0.52 (0.33–0.74)	0.81 (0.57–1.12)	0.82 (0.27–2.25)	0.88 (0.46–1.54)
Georges River	0.78 (0.36–1.39)	0.95 (0.65–1.34)	0.70 (0.24–1.92)	1.50 (0.60–2.99)
UNSCA	0.76 (0.47–1.14)	1.52 (1.04–2.14)	0.53 (0.18–1.45)	0.93 (0.43–1.74)
Southern Tablelands	0.61 (0.42–0.84)	0.94 (0.67–1.29)	0.68 (0.22–1.89)	0.81 (0.47–1.35)

Values represent debiased mean ratios with 95% confidence intervals estimated using hierarchical 𝜒^2^ inverse Gaussian meta-analysis. Confidence intervals not overlapping 1 indicate statistically significant differences.

Despite substantial variation in population-level home range size and diffusion rates among our study regions (Fig. 2), the ratio of male – female home range sizes and diffusion rates within each population were markedly consistent. Male home ranges were typically twice as large as females in each region (Table 4), with all regions except for Richmond Range and UNSCA (the two regions with the smallest sample sizes of males) having effect size ratio confidence intervals not overlapping one. Male diffusion rates were generally two-to-four-fold greater than females, with only Port Macquarie male – female diffusion rate effect size ratio confidence intervals marginally overlapping one (Table 4).

**Table 4:** Within region effect size ratios for male – female home range areas and diffusion rates

Region	M: F Home Range Size Ratio (95% CI)	M: F Diffusion Rate Ratio (95% CI)
Port Macquarie	2.44 (1.19–4.35)	2.13 (0.93–3.98)
Narrandera	2.01 (1.18–3.28)	5.99 (3.49–9.67)
Richmond Range	1.80 (0.95–3.35)	2.84 (1.62–4.59)
Kanangra-Boyd	2.05 (1.24–3.26)	4.06 (2.12–6.94)
Georges River	2.58 (1.41–4.20)	4.47 (1.70–8.66)
UNSCA	1.43 (0.69–2.73)	3.62 (1.81–6.51)
Southern Tablelands	2.20 (1.37–3.28)	3.47 (2.43–4.76)

Values represent debiased mean ratios with 95% confidence intervals estimated using hierarchical 𝜒^2^ inverse Gaussian meta-analysis [44]. Confidence intervals not overlapping 1 indicate statistically significant differences

## Discussion

This study is the first application of continuous-time movement modelling for koalas at scale, providing robust estimates of fundamental movement parameters across a broad range of environmental conditions. By analysing GPS telemetry data from 230 koalas spanning seven populations, we demonstrated the underlying temporal structure of koala movement behaviour is remarkably conserved despite substantial variation in the spatial extent of their movements. Position and velocity autocorrelation timescales – representing range crossing time and directional persistence of motion respectively – showed relatively little variation across populations despite 7-10-fold differences in home range size and diffusion rates. Similarly, the magnitude of sexual dimorphism in space use and movement behaviour was consistent across all study regions; males maintained approximately twice the home range area and moved more (two to six times the diffusion rate) than females each day. Importantly, all koala movement datasets exhibited varying degrees of autocorrelation, even when sampled relatively infrequently (e.g. 12-hourly). These findings suggest that certain aspects of koala movement ecology are fundamental characteristics of the species, with important implications for the design of both telemetry studies and broader monitoring programs.

We observed substantial variation in home range size among our study regions, consistent with the broad range of estimates reported for koalas across their distribution (e.g., [16, 28, 49–51]). Previous studies have documented koala home ranges varying from less than two hectares in high-quality coastal habitat to over 100 hectares in semi-arid environments, with this variation often attributed to differences in habitat quality and resource availability [52–54]. Our results are consistent with this pattern: koalas in Narrandera and Port Macquarie occupy habitats dominated by high quality forage species such as *Eucalyptus camaldulensis*, *E. tereticornis*, *E. microcorys* and *E. robusta* in productive riparian and coastal environments. In contrast, populations in the Southern Tablelands and UNSCA occupy drier, more heterogenous landscapes where availability of resources is likely more patchily distributed. Individual heterogeneity in home range size within regions was also moderate (Table S5), presumably reflecting fine-scale variation in habitat quality and resource availability [55] alongside intrinsic traits such as individual condition, sex and age [56, 57]. Explicitly testing drivers of variation in space use at either scale (e.g., [32, 58]), whilst worthwhile, was beyond the scope of this study. Nevertheless, the substantial regional and individual variation in space use we demonstrate has important implications for koala conservation planning. Habitat restoration, revegetation corridor design, vehicle strike mitigation and protected area planning risk being ineffective if based on space use from estimates from geographically or ecologically distant populations. Instead, effective koala conservation strategies require locally calibrated management strategies that account for population-specific movement scales and habitat requirements [59–61].

Despite a broad gradient of home range sizes and diffusion rates, the temporal structure of koala movement was much less variable among our study regions. Except for Narrandera, range crossing times were broadly similar across regions for both males and females (Fig. 2). That koalas in small, resource-rich home ranges cross their range on similar timescales to those in large and potentially resource-poor ranges suggests that movement rate scales with range size. That is, animals with larger ranges move throughout a larger area each day yet revert to their mean location at relatively consistent timescales. For males, this pattern may reflect the demands of territorial maintenance and scent-marking where regular traversal of the home range is necessary to maintain territorial signalling regardless of absolute range size ([62–64]). The functional basis for similar range crossing times in females with varying home range sizes is less clear, though it may similarly reflect constraints on how frequently koalas need to move throughout their range to meet ecological or social requirements [65–67]. Koala spatial organisation typically entails broadly overlapping home range areas yet minimal resource sharing [65]. This pattern of overlapping but generally spatially segregated home ranges may necessitate regular range traversal to maintain awareness of neighbouring individuals and avoid direct competition for resources, even when overall range sizes vary considerably. This may also explain why both male and female koalas in Narrandera moved notably faster throughout their range relative to other regions with marginally smaller or larger population level home range sizes such as Port Macquarie and Richmond Range (Fig. 2). Concurrent drone surveys in Narrandera conducted annually during our study indicate that this region has the highest density of koalas among our study regions (Jessop et al., unpublished data). We emphasise, however, that the relationship between density and range-crossing time is not directly tested here. Instead, this explanation is offered as a hypothesis consistent with the observed pattern and consequently, additional studies quantifying environmental and social correlates of range crossing time are needed to better understand the underlying causal mechanisms driving variation in koala movement behaviour.

Directional persistence timescales were also broadly similar across regions for both males and females, with largely overlapping confidence intervals among most populations (Fig. 2). This timescale aligns well with fine-scale observations of koala movements where koalas typically move between trees in discrete bouts before settling into extended sedentary periods of feeding or rest [68–70]. This suggests that velocity autocorrelation captures a fundamental unit of koala movement behaviour – the inter-tree movement bout – that operates on similar timescales regardless of home range size. Whilst the temporal duration of these movement bouts is conserved among regions, the actual distances traversed during these periods of directed motion (i.e. the ballistic length scale, [71]) likely varies considerably, presumably scaling with home range size, tree density and landscape configuration [29, 53, 72]. One implication of this pattern is that koalas with smaller ranges in more productive habitats may have greater opportunity to assess and discriminate among potential destination trees within a given movement bout. Whether differences in directional persistence timescales and actual distance traversed translates to different resource selection behaviours when moving between trees warrants further investigation and could be validated by pairing fine-scale telemetry data with intensive field observations of koala behaviour when moving between trees (e.g., [68, 70]).

A key finding of this study was the remarkably consistent sexual dimorphism in space use across all study regions. Irrespective of local habitat conditions, male koalas maintained home ranges approximately twice the size of females and moved throughout broader areas relative to females on a daily basis (Fig. 2; Table 4). That male home ranges are larger than females is well established in the literature and this behaviour is typically attributed to the koala’s polygynous mating system wherein males range more widely to encompass the home ranges of multiple females [16, 65, 73]. However, that the magnitude of this difference remains constant across such a broad environmental gradient has not been previously documented. This consistency suggests that the scaling relationship between male and female space use is a fundamental feature of koala social organisation that operates independently of local habitat conditions.

Whilst there are few examples in the literature to compare to, the broadly invariant range crossing time and directional persistence timescales we observed across koala populations align with patterns reported for lowland tapirs (*Tapirus terrestris*) across three distinct Brazilian biomes, where, [32] similarly found that range crossing time and directional persistence showed little variation despite substantial differences in habitat composition and human disturbance. In contrast, [33] estimated range crossing times and directional persistence of white-bearded wildebeest (*Connochaetes taurinus*) across three East African grazing systems and demonstrated animals living in areas with higher anthropogenic disturbance had restricted movement relative to those living in more contiguous habitat. Whether range crossing time and directional persistence is conserved or plastic across populations is likely species- and context-specific, and more studies are needed across taxa to understand when and why the temporal structure of movement varies.

### Monitoring implications

The following recommendations follow from the movement parameters estimated here rather than from explicit evaluation within this study, and are intended as considerations for the design of future koala telemetry and monitoring programs rather than as protocols validated by the current dataset.

The consistency of autocorrelation timescales across our study regions has important implications for the design of GPS telemetry studies in koalas. Because range crossing time and directional persistence timescales were largely invariant to habitat type and regional differences in space use, optimal sampling schedules derived from one population should be broadly transferable to others across the species’ range in New South Wales. This simplifies study design considerably: researchers planning koala telemetry studies may not need to trial different fix intervals for coastal versus inland or tablelands populations, as the underlying temporal structure of koala movement behavior appears similar regardless of local conditions. The CTMM framework provides explicit guidance on sampling requirements relative to these timescales: to reliably estimate home ranges, deployment durations should be long enough to allow animals to move throughout their home range several times [14, 39]. For koalas, this suggests minimum deployments of several weeks to months depending on sex, the precision required, and research objectives.

Importantly, the slow range-crossing time of koalas suggests that changes in home range size or location may be an insensitive metric for assessing koala responses to management-relevant perturbations such as prescribed fire, translocation or logging. Detecting real shifts in space use requires monitoring windows long enough to capture multiple complete range traversals both before and after the perturbation of interest. If animals require several weeks to move throughout their range under normal conditions, home range estimates derived from short monitoring windows will be based on incomplete sampling of the underlying movement process. Whilst methods such as AKDE can still be applied to highly autocorrelated data within short time windows, the resulting estimates will have low effective sample sizes and correspondingly wide confidence intervals, making statistical inference about changes in space use challenging ([14, 39]). This has particular relevance for translocation programs, where confirming that an animal has “settled” into a stable home range post-release is often a key indicator of success (e.g., [74, 75]). Our data suggests that establishing settlement of translocated koalas with confidence would require extended post-release monitoring (e.g. several months) before range residency can be reliably determined, particularly if exploratory movements post-release are maintained for an extended time. Where monitoring windows are short relative to range crossing times, parametric bootstrapping methods can improve home range estimation [39]. However, such corrections cannot always compensate for fundamentally inadequate sampling durations. Alternatively, approaches that are less reliant on defining the full utilization distribution such as estimating distance traversed [6] or identifying when aspects of the underlying movement process change [10] may provide more sensitive metrics for detecting how koalas respond to stochastic events or management interventions. Irrespective of analytical objectives, given the slow range crossing times of koalas, we recommend koala telemetry studies systematically report individual survey durations alongside nominal sample sizes of locations collected when estimating koala home ranges.

The slow range crossing time of koalas also has implications for resource selection analyses. Resource selection functions (RSFs) compare environmental conditions at used locations with those sampled from an availability domain, typically defined by the animal’s home range estimated from the full tracking period (Boyce 2006; [76]). However, RSF coefficients are highly sensitive to how availability is defined [76–78]. When GPS data are collected at intervals shorter than the range crossing time, the true availability domain accessible to an animal is a function of both its current position and its movement capacity [7]. Consequently, when the movement process is sampled more frequently than the range crossing time, deriving available locations from the long-term utilization distribution results in locations that are deemed available yet are fundamentally unrealistic given the movement behavior of the animal. This mismatch compromises inference as it can result in selection coefficients that are biased and overly confident [7]. Indeed, our results suggest extensive thinning of koala movement data may be required to meet the statistical independence assumptions of discrete-time RSFs for koalas. Step-selection functions (SSFs) offer a powerful alternative approach to assessing resource selection in animal movement, as they explicitly define availability as conditional on the animal’s previous location and movement characteristics [79, 80]. Despite these analytical advantages, to the best of our knowledge, there are no examples of SSFs fitted to koala telemetry data in the literature. Nevertheless, if fitting RSFs to koala movement data is a key research objective, then our results suggest movement data would need to be thinned to intervals on the order of 3–5-day intervals, otherwise integrated approaches that explicitly account for autocorrelation when defining availability are required [7].

The short velocity autocorrelation timescales we observed have implications for research objectives that depend on resolving fine-scale movement behaviour. Studies investigating encounter rates between animals increasingly rely on continuous-time approaches to estimate when and where potential contact events occur from GPS tracking data [81–84]. These approaches are well suited to understanding how animal movement influences disease transmission dynamics. For example, [85] used continuous-time movement models to estimate spatially explicit interaction networks for disease systems including African swine fever in wild pigs and chronic wasting disease in mule deer. However, simulations have demonstrated that encounter rates derived from GPS data can be substantially underestimated when the temporal resolution of movement data exceeds 30-minute intervals [85, 86]. With sampling regimes of two, four or six hours – common in many contemporary koala telemetry studies (e.g., [28, 72, 87]) – path reconstruction methods effectively interpolate across what were likely several discrete movements interspersed with sedentary periods, potentially misrepresenting encounter probabilities and the spatial distribution of contact events. This is of direct relevance to koalas, as *Chlamydia pecorum* infection remains the most significant infectious disease threat across the species’ distribution [24, 61, 88]. Given that chlamydial transmission occurs through close contact during mating, mother-offspring interactions, and potentially shared tree use [89–91], our data suggest high resolution sampling (e.g. 30 min or less) would likely be required to accurately resolve when and where koalas encounter each other.

Estimates of daily distance travelled or movement speeds can also benefit from considering velocity autocorrelation timescales. Traditional approaches that sum straight-line displacements between locations are highly sensitive to the sampling frequency, with coarser sampling systematically underestimating true path length as the tortuous fine-scale structure of movement is not captured [6]. For example, studies in primates have demonstrated that moving from sub-minute to hourly GPS fixes can reduce estimated daily travel distances by over 60% [92]. Whilst continuous-time methods for speed and distance estimation can correct for these biases provided the sampling interval doesn’t exceed approximately three times Ƭ_v_ [6], many koala telemetry studies operate at sampling intervals far exceeding this threshold. Consequently, research questions requiring accurate estimates of daily movement distances as a proxy for cumulative energy expenditure in koalas will also benefit from data collected at frequent intervals (i.e. three hourly or less) during active periods.

Beyond telemetry studies, the autocorrelation time scales we estimated can inform the design and interpretation of other monitoring approaches increasingly applied to koala populations. For example, thermal drone surveys are now widely used for koala monitoring across eastern Australia [93–95]. Analysts often use N-mixture models to estimate abundance (e.g. 96, 97, 98]) and these models typically assume site closure across replicate survey occasions within a sampling session (i.e. no births, deaths, emigration or immigration during the survey period) [99, 100]. Whilst the assumption of no births or deaths is generally plausible for short survey periods (e.g. days to weeks), increasing attention has been paid to the influence of movement of individuals into and out of the survey plot (temporary emigration) between replicate surveys when fitting N-mixture models [101–103]. The likelihood of temporary emigration between occasions likely depends on many factors, such as a species’ movement rate, the size of their range relative to survey plot dimensions, the spatial arrangement of ranges throughout the broader survey area, and the time interval between replicate surveys [104]. While temporary emigration can be accommodated if it occurs randomly [101, 105], abundance estimates derived under conditions where the closure assumption is likely violated better represent the “superpopulation” – the number of animals who could have used the plot area during the survey period – rather than instantaneous abundance.

This is an important distinction to consider when comparing abundance estimates derived from populations with different home range sizes, survey periods and spatial arrangements of drone plots. For example, consider a drone survey design with a fixed plot size (e.g. 25 hectares) and conducted over a long enough period wherein animals can move throughout the entirety of their home range and temporary emigration likely occurs between replicate surveys (e.g. two weeks). In a region where ranges are smaller than drone plots this superpopulation may incorporate animals living only a short distance beyond plots. Correspondingly, drone plots separated by a distance of several home range diameters are likely statistically independent and sampling from non-overlapping superpopulations. Conversely, if ranges are large relative to drone plots, the superpopulation likely includes animals living well beyond the plot area and even though sites may be geographically separated by several hundred meters, they may be sampling from a similar pool of individuals and therefore spatially autocorrelated. Fortunately, the generally invariant range crossing times of koalas irrespective of home range size (Fig. 2) suggests that drone survey designs characterised by short total survey periods (< 3 days) and minimal time between replicate flights should be more likely to meet closure assumptions when fitting N-mixture models.

### Limitations

Several limitations should be acknowledged when interpreting our findings. First, sample sizes of range-resident animals were limited in some regions, particularly males in Richmond Range and Georges River (Table 2). This resulted in greater uncertainty in population-level parameter estimates for these sites (Fig. 2, Table S5). Relatedly, the exclusion of transient individuals means that our regional estimates characterise the range-resident cohort of each population rather than all collared animals, and all among-region comparisons are therefore conditional on range residency. Transient behaviour may itself reflect genuine ecological differences among regions such as resource quality and stochasticity [106]. Correspondingly, characterising the drivers and prevalence of transient movement behaviour across populations is a worthwhile avenue for future work.

Second, despite extended monitoring periods across all regions, the slow range-crossing times of koalas resulted in modest effective sample sizes for home range estimation (mean range: 16–54, Table S4). Correspondingly, most individual-level home range estimates were associated with moderate uncertainty even though nominal sample sizes were often in the order of 100’s to 1000’s of locations. Effective sample sizes could have been increased by adopting a less stringent definition of the breeding season and incorporating data from other months into our analyses (e.g. December, January and August). However, we deliberately excluded these months to avoid potential biases in home range size estimates arising from altered movement behaviour during the breeding season (e.g., increased male ranging, [49]), prioritizing the characterization of baseline, non-breeding movement patterns. This conservative approach sacrificed some statistical precision in exchange for reduced risk of confounding seasonal behavioural changes with routine space use.

Third, while our dataset encompassed seven populations spanning a broad environmental gradient across NSW, this represents a limited spatial sample of the koala’s overall distribution that does not capture the extremes of koala space use documented elsewhere (e.g., [51, 52]). It’s possible that range crossing times and directional persistence timescales where koala ranges are very small or very large may differ from those presented here, potentially reflecting fundamentally different movement strategies when resources are highly concentrated or widely dispersed.

Finally, our study sites were comprised of largely contiguous native vegetation blocks. It is possible that koala movement in fragmented or peri-urban environments may also differ in fundamental ways to that observed in this study (e.g., [18, 54, 107]), with implications for range crossing times and directional persistence that warrant further study.

## Conclusions

Understanding animal movement through the lens of autocorrelation timescales offers fundamental insights into how species perceive and interact with their environment [4, 56]. This study represents the first application of continuous-time movement models to koalas at scale, providing empirical estimates of movement timescales and other commonly used movement metrics for a species of significant conservation concern.

Tools now exist to evaluate sampling design decisions a priori given knowledge of a species’ movement timescales, including the *movedesign* application which enables researchers to assess the precision achievable under different sampling regimes [15]. We encourage practitioners to use these tools and the parameter estimates derived in our study when designing koala telemetry studies. Additionally, the estimates of fundamental movement properties we derived are well suited as empirical inputs for simulation research. For instance, agent-based models are a powerful tool for exploring a broad range of ecological research and management questions (e.g. [108, 109]). The baseline movement parameters established here could enable virtual experiments of different management or disease transmission scenarios with realistic movement rules rather than assumed values.

Whilst technological advances allow researchers to collect data more frequently and for longer periods, accounting for autocorrelation in movement data is critical for reducing bias in parameter estimates and improving the reliability of management recommendations [8]. The autocorrelation-informed weighting approaches developed for home range estimation [14, 43], resource selection [7] and distance travelled [6] clearly demonstrate how accounting for the temporal structure of movement data improves inference. For koalas, we encourage matching sampling design to species-specific timescales of movement and the behavior of interest to improve the reliability of telemetry-derived management recommendations used in conservation planning.

## Supplementary Information

Below is the link to the electronic supplementary material.

Supplementary Material 1

## Data Availability

The datasets supporting the conclusions of this article will be made available on the NSW government’s Sharing and Enabling Environmental Data (SEED; https://www.seed.nsw.gov.au/) repository by September 2026.

## References

[1] Kays R, Crofoot MC, Jetz W, Wikelski M. Terrestrial animal tracking as an eye on life and planet. Science. 2015;348(6240):2478. 10.1126/science.aaa2478 26068858

[2] Nathan R, Monk CT, Arlinghaus R, Adam T, Alós J, Assaf M, Baktoft H, Beardsworth CE, Bertram MG, Bijleveld AI, Brodin T. Big-data approaches lead to an increased understanding of the ecology of animal movement. Science. 2022;375(6582):eabg1780. 10.1126/science.abg1780 35175823

[3] Hampton SE, Strasser CA, Tewksbury JJ, Gram WK, Budden AE, Batcheller AL, Duke CS, Porter JH. Big data and the future of ecology. Front Ecol Environ. 2013;11(3):156–62.

[4] Fleming CH, Calabrese JM, Mueller T, Olson KA, Leimgruber P, Fagan WF. From fine-scale foraging to home ranges: a semivariance approach to identifying movement modes across spatiotemporal scales. Am Nat. 2014;183(5):E154–67. 10.1086/675504 24739204

[5] Fleming CH, Fagan WF, Mueller T, Olson KA, Leimgruber P, Calabrese JM. Rigorous home range estimation with movement data: a new autocorrelated kernel density estimator. Ecology. 2015;96(5):1182–8. 10.1890/14-2010.1 26236833

[6] Noonan MJ, Fleming CH, Akre TS, Drescher-Lehman J, Gurarie E, Harrison AL. Scale-insensitive estimation speed distance traveled Anim Track data Mov Ecol. 2019a;7:35. 10.1186/s40462-019-0177-1 PMC6858693 31788314

[7] Alston JM, Fleming CH, Kays R, Streicher JP, Downs CT, Ramesh T, Reineking B, Calabrese JM. Mitigating pseudoreplication and bias in resource selection functions with autocorrelation-informed weighting. Methods Ecol Evol. 2023;14(2):643–54.

[8] Noonan MJ, Tucker MA, Fleming CH, Akre TS, Alberts SC, Ali AH, Altmann J, Antunes PC, Belant JL, Beyer D, Blaum N. A comprehensive analysis of autocorrelation and bias in home range estimation. Ecol Monogr. 2019b;89(2):e01344.

[9] Calabrese JM, Fleming CH, Gurarie E. ctmm: an R package for analyzing animal relocation data as a continuous-time stochastic process. Methods Ecol Evol. 2016;7(9):1124–32.

[10] Gurarie E, Fleming CH, Fagan WF, Laidre KL, Hernández-Pliego J, Ovaskainen O. Correlated velocity models as a fundamental unit of animal movement: synthesis and applications. Mov Ecol. 2017;5(1):13. 10.1186/s40462-017-0103-3 PMC5424322 28496983

[11] Johnson DS, London JM, Lea MA, Durban JW. Continuous-time correlated random walk model for animal telemetry data. Ecology. 2008;89(5):1208–15. 10.1890/07-1032.1 18543615

[12] Fleming CH, Drescher-Lehman J, Noonan MJ, Akre TSB, Brown DJ, Cochrane MM, Dejid N, DeNicola V, DePerno CS, Dunlop JN, Gould NP. A comprehensive framework for handling location error in animal tracking data. BioRxiv. 2020; p. 2020–06.

[13] Fleming CH, Calabrese JM. A new kernel density estimator for accurate home-range and species‐range area estimation. Methods Ecol Evol. 2017;8(5):571–9.

[14] Silva I, Fleming CH, Noonan MJ, Alston J, Folta C, Fagan WF, Calabrese JM. Autocorrelation-informed home range estimation: A review and practical guide. Methods Ecol Evol. 2022;13(3):534–44.

[15] Silva I, Fleming CH, Noonan MJ, Fagan WF, Calabrese JM. movedesign: Shiny R app to evaluate sampling design for animal movement studies. Methods Ecol Evol. 2023;14(9):2216–25.

[16] Ellis WAH, Melzer A, Carrick FN, Hasegawa M. Tree use, diet and home range of the koala (Phascolarctos cinereus) at Blair Athol, central Queensland. Wildl Res. 2002;29(3):303–11.

[17] Marsh KJ, Wallis IR, Foley WJ. Behavioural contributions to the regulated intake of plant secondary metabolites in koalas. Oecologia. 2007;154(2):283–90. 10.1007/s00442-007-0828-6 17690913

[18] McAlpine CA, Rhodes JR, Callaghan JG, Bowen ME, Lunney D, Mitchell DL, Pullar DV, Possingham HP. The importance of forest area and configuration relative to local habitat factors for conserving forest mammals: a case study of koalas in Queensland, Australia. Biol Conserv. 2006;132(2):153–65.

[19] Rhodes JR, Wiegand T, McAlpine CA, Callaghan J, Lunney D, Bowen M, Possingham HP. Modeling species’ distributions to improve conservation in semiurban landscapes: koala case study. Conserv Biol. 2006;20(2):449–59. 10.1111/j.1523-1739.2006.00330.x 16903106

[20] Dique DS, Thompson J, Preece HJ, Penfold GC, Villiers DLD, Leslie RS. Koala mortality on roads in south-east Queensland: the koala speed-zone trial. Wildl Res. 2003;30(4):419–26.

[21] Gonzalez-Astudillo V, Henning J, Valenza L, Knott L, McKinnon A, Larkin R, Allavena R. A necropsy study of disease and comorbidity trends in morbidity and mortality in the koala (Phascolarctos cinereus) in South-East Queensland, Australia. Sci Rep. 2019;9(1):17494. 10.1038/s41598-019-53970-0 PMC6877607 31767897

[22] Gonzalez-Astudillo V, Allavena R, McKinnon A, Larkin R, Henning J. Decline causes of Koalas in South East Queensland, Australia: a 17-year retrospective study of mortality and morbidity. Sci Rep. 2017;7(1):42587. 10.1038/srep42587 PMC5316976 28218272

[23] McCallum H, Kerlin DH, Ellis W, Carrick F. Assessing the significance of endemic disease in conservation—koalas, chlamydia, and koala retrovirus as a case study. Conserv Lett. 2018;11(4):e12425.

[24] Vitali SD, Reiss AE, Jakob-Hoff RM, Stephenson TL, Holz PH, Higgins DP. National koala disease risk analysis report V 1.2. National Koala Disease Risk Analysis Report V 1.2. 2023. 10.25910/xkkv-nk38.

[25] Adams-Hosking C, Grantham HS, Rhodes JR, McAlpine C, Moss PT. Modelling climate-change-induced shifts in the distribution of the koala. Wildl Res. 2011;38(2):122–30.

[26] Ellis W, Melzer A, Clifton I, Carrick F. Climate change and the koala Phascolarctos cinereus: water and energy. Australian Zoologist. 2010;35(2):369–77.

[27] Bista D, Allen B, Baxter G, Booth R, Reardon-Smith K, Gorecki V, Murray P. Immediate impacts of fire on koala movement in a fragmented landscape. Global Ecol Conserv. 2024;56:e03274.

[28] Law B, Gonsalves L, Slade C, Brassil T, Flanagan C. GPS tracking reveals koalas Phascolarctos cinereus use mosaics of different forest ages after environmentally regulated timber harvesting. Austral Ecol. 2024;49(4):e13518.

[29] Tacla PK, Barth BJ, FitzGibbon SI, Gillett AK, Ellis WA. Patterns of activity and travel by koalas in a disturbed urban landscape in Queensland. Australian Mammalogy. 2025;47(1). 10.1071/AM24044.

[30] Radford SL, McKee JEFF, Goldingay RL, Kavanagh RP. The protocols for koala research using radio-collars: a review based on its application in a tall coastal forest in New South Wales and the implications for future research. Australian Mammalogy. 2006;28(2):187–200.

[31] Hebblewhite M, Haydon DT. Distinguishing technology from biology: a critical review of the use of GPS telemetry data in ecology. Philosophical Trans Royal Soc B: Biol Sci. 2010;365(1550):2303–12. 10.1098/rstb.2010.0087 PMC2894965 20566506

[32] Medici EP, Mezzini S, Fleming CH, Calabrese JM, Noonan MJ. Movement ecology of vulnerable lowland tapirs between areas of varying human disturbance. Mov Ecol. 2022;10(1):14. 10.1186/s40462-022-00313-w PMC8919628 35287742

[33] Stabach JA, Hughey LF, Crego RD, Fleming CH, Hopcraft JGC, Leimgruber P, Morrison TA, Ogutu JO, Reid RS, Worden JS, Boone RB. Increasing anthropogenic disturbance restricts wildebeest movement across East African grazing systems. Front Ecol Evol. 2022;10:846171.

[34] Hanger J, Gillett A. Veterinary involvement in the research and management of free-ranging koala populations. In: Vogelnest L, Portas T, editors. Current Therapy in Medicine of Australian Mammals. Melbourne: CSIRO Publishing; 2025. 10.1071/9781486307524.BK07710_ch35.

[35] Madani GF, Ashman KR, Mella VSA, Whisson DA. A review of the ‘noose and flag’ method to capture free-ranging koalas. Australian Mammalogy. 2020;42(3):341–8.

[36] Ellis W, Bercovitch F, Fitzgibbon S, Roe P, Wimmer J, Melzer A, Wilson R. Koala bellows and their association with the spatial dynamics of free-ranging koalas. Behav Ecol. 2011;22(2):372–7.

[37] Martin R, Handasyde KA. The koala: natural history, conservation and management. UNSW; 1999.

[38] Ponchon A. Prospecting for informed dispersal. reappraisal of a widespread but overlooked ecological process; 2026. 10.1002/ecy.70411 42169628

[39] Fleming CH, Noonan MJ, Medici EP, Calabrese JM. Overcoming the challenge of small effective sample sizes in home-range estimation. Methods Ecol Evol. 2019;10(10):1679–89.

[40] Burnham KP, Anderson DR. Multimodel inference: understanding AIC and BIC in model selection. Sociol methods Res. 2004;33(2):261–304.

[41] Uhlenbeck GE, Ornstein LS. On the theory of the Brownian motion. Phys Rev. 1930;36(5):823.

[42] DeNicola VL, Mezzini S, Cagnacci F, Fleming CH. Are your data too coarse for speed estimation? Diffusion rates as an alternative measure of animal movement. bioRxiv. 2025;2025–07. 10.1101/2025.07.17.665364.

[43] Fleming CH, Sheldon D, Fagan WF, Leimgruber P, Mueller T, Nandintsetseg D, Noonan MJ, Olson KA, Setyawan E, Sianipar A, Calabrese JM. Correcting for missing and irregular data in home-range estimation. Ecol Appl. 2018;28(4):1003–10. 10.1002/eap.1704 29450936

[44] Fleming CH, Deznabi I, Alavi S, Crofoot MC, Hirsch BT, Medici EP, Noonan MJ, Kays R, Fagan WF, Sheldon D, Calabrese JM. Population-level inference for home‐range areas. Methods Ecol Evol. 2022;13(5):1027–41.

[45] Silva I, Fleming CH, Noonan MJ, Fagan WF and Calabrese JM. Too few, too many, or just right? Optimizing sample sizes for population‐level inferences in animal tracking projects. Ecol Evol. 2026;16(6):e73755. 10.1002/ece3.73755. PMC13239661 42256161

[46] Sullivan GM, Feinn R. Using effect size—or why the P value is not enough. J graduate Med Educ. 2012;4(3):279–82. 10.4300/JGME-D-12-00156.1 PMC3444174 23997866

[47] R Core Team. R: A Language and Environment for Statistical Computing. Vienna, Austria: R Foundation for Statistical Computing; 2023. https://www.R-project.org/.

[48] Fleming CH, Calabrese JM. ctmm: Continuous-Time Movement Modeling. R package version 1.3.1. 2025. https://github.com/ctmm-initiative/ctmm

[49] Ashman KR, Page NR, Whisson DA. Ranging behavior of an arboreal marsupial in a plantation landscape. J Wildl Manag. 2020;84(6):1091–9.

[50] Goldingay RL, Dobner B. Home range areas of koalas in an urban area of north-east New South Wales. Australian Mammalogy. 2014;36(1):74–80.

[51] Whisson DA, Dixon V, Taylor ML, Melzer A. Failure to respond to food resource decline has catastrophic consequences for koalas in a high-density population in southern Australia. PLoS ONE. 2016;11(1):e0144348. 10.1371/journal.pone.0144348 PMC4703219 26735846

[52] Davies N, Gramotnev G, Seabrook L, Bradley A, Baxter G, Rhodes J, Lunney D, McAlpine C. Movement patterns of an arboreal marsupial at the edge of its range: a case study of the koala. Mov Ecol. 2013;1(1):8. 10.1186/2051-3933-1-8 PMC4337771 25709822

[53] Rus AI, McArthur C, Mella VS, Crowther MS. Habitat fragmentation affects movement and space use of a specialist folivore, the koala. Anim Conserv. 2021;24(1):26–37.

[54] Whisson DA, Zylinski S, Ferrari A, Yokochi K, Ashman KR. Patchy resources and multiple threats: How do koalas navigate an urban landscape? Landsc Urban Plann. 2020;201:103854.

[55] Moore BD, Foley WJ. A review of feeding and diet selection in koalas (Phascolarctos cinereus). Australian J Zool. 2000;48(3):317–33.

[56] Nathan R, Getz WM, Revilla E, Holyoak M, Kadmon R, Saltz D, Smouse PE. A movement ecology paradigm for unifying organismal movement research. Proc Natl Acad Sci. 2008;105(49):19052–9. 10.1073/pnas.0800375105 PMC2614714 19060196

[57] White NA. Ecology of the koala (Phascolarctos cinereus) in rural south-east Queensland, Australia. Wildl Res. 1999;26(6):731–44.

[58] Brown MB, Fennessy JT, Crego RD, Fleming CH, Alves J, Brandlová K, Fennessy S, Ferguson S, Hauptfleisch M, Hejcmanova P, Hoffman R. Ranging behaviours across ecological and anthropogenic disturbance gradients: a pan-African perspective of giraffe (Giraffa spp.) space use. Proc R Soc B. 2023;290(2001). 10.1098/rspb.2023.0912 PMC10291724 37357852

[59] Ashman KR, Watchorn DJ, Whisson DA. Prioritising research efforts for effective species conservation: a review of 145 years of koala research. Mammal Rev. 2019;49(2):189–200.

[60] McAlpine C, Lunney D, Melzer A, Menkhorst P, Phillips S, Phalen D, Ellis W, Foley W, Baxter G, De Villiers D, Kavanagh R. Conserving koalas: a review of the contrasting regional trends, outlooks and policy challenges. Biol Conserv. 2015;192:226–36.

[61] Rhodes JR, Ng CF, de Villiers DL, Preece HJ, McAlpine CA, Possingham HP. Using integrated population modelling to quantify the implications of multiple threatening processes for a rapidly declining population. Biol Conserv. 2011;144(3):1081–8.

[62] Salamon M, Davies NW, Stoddart DM. Olfactory communication in Australian marsupials with particular reference to brushtail possum, koala, and eastern grey kangaroo. Advances in chemical signals in vertebrates. Boston, MA: Springer US; 1999. pp. 85–98. 10.1007/978-1-4615-4733-4_6.

[63] Gosling LM, Roberts SC. Scent-marking by male mammals: cheat-proof signals to competitors and mates. Advances in the Study of Behavior. Vol. 30. Academic; 2001. p. 169–217. 10.1016/S0065-3454(01)80007-3.

[64] Charlton BD. Chemosensory discrimination of identity and familiarity in koalas. Behav Process. 2015;119:38–43. 10.1016/j.beproc.2015.07.008 26216198

[65] Ellis WA, Melzer A, Bercovitch FB. Spatiotemporal dynamics of habitat use by koalas: the checkerboard model. Behav Ecol Sociobiol. 2009;63(8):1181–8.

[66] Mitchell P. The home ranges and social activity of koalas: a quantitative analysis. In: Lee AK, Handasyde KA, Sanson GD, editors. Biology of the Koala. Sydney: Surrey Beatty & Sons; 1990. p. 171–187.

[67] Russell EM. Social behaviour and social organization of marsupials. Mammal Rev. 1984;14(3):101–54.

[68] Marsh KJ, Moore BD, Wallis IR, Foley WJ. Continuous monitoring of feeding by koalas highlights diurnal differences in tree preferences. Wildl Res. 2014;40(8):639–46.

[69] Ryan MA, Whisson DA, Holland GJ, Arnould JP. Activity patterns of free-ranging koalas (Phascolarctos cinereus) revealed by accelerometry. PLoS ONE. 2013;8(11):e80366. 10.1371/journal.pone.0080366 PMC3817117 24224050

[70] Sparkes GR, Wilson O, Ellis WA, FitzGibbon SI, Barth BJ, Clemente CJ, Crowther MS, Wilson RS. Between the Trees: Quantifying Koala Ground Movement for Conservation Action. Animals. 2025;15(24):3537. 10.3390/ani15243537 PMC12729488 41463822

[71] Noonan MJ, Martinez-Garcia R, Fleming CH, De Figueiredo BG, Ali AH, Attias N, Belant JL, Beyer DE Jr, Berteaux D, Bidner LR, Boone R. The search behavior of terrestrial mammals. BioRxiv. 2023. p. 2022-12.

[72] Crowther MS, Rus AI, Mella VS, Krockenberger MB, Lindsay J, Moore BD, McArthur C. Patch quality and habitat fragmentation shape the foraging patterns of a specialist folivore. Behav Ecol. 2022;33(5):1007–17. 10.1093/beheco/arac068 PMC9639584 36382228

[73] Gordon G, McGreevy DG, Lawrie BC. Koala population turnover and male social organization. In: Lee AK, Handasyde KA, Sanson GD, editors. Biology of the Koala. 1990. p. 189–192.

[74] Leigh KA, Hofweber LN, Sloggett BK, Inman VL, Pettit LJ, Sriram A, Haering R. Outcomes for an arboreal folivore after rehabilitation and implications for management. Sci Rep. 2023;13(1):6542. 10.1038/s41598-023-33535-y PMC10121558 37085564

[75] Menkhorst P, Ramsey D, O’Brien T, Hynes E, Whisson D. Survival and movements of koalas translocated from an over-abundant population. Wildl Res. 2019;46(7):557–65.

[76] Northrup JM, Hooten MB, Anderson CR Jr, Wittemyer G. Practical guidance on characterizing availability in resource selection functions under a use–availability design. Ecology. 2013;94(7):1456–63. 10.1890/12-1688.1 23951705

[77] Beyer HL, Haydon DT, Morales JM, Frair JL, Hebblewhite M, Mitchell M, Matthiopoulos J. The interpretation of habitat preference metrics under use–availability designs. Philosophical Trans Royal Soc B: Biol Sci. 2010;365(1550):2245–54. 10.1098/rstb.2010.0083 PMC2894962 20566501

[78] Forester JD, Im HK, Rathouz PJ. Accounting for animal movement in estimation of resource selection functions: sampling and data analysis. Ecology. 2009;90(12):3554–65. 10.1890/08-0874.1 20120822

[79] Avgar T, Potts JR, Lewis MA, Boyce MS. Integrated step selection analysis: bridging the gap between resource selection and animal movement. Methods Ecol Evol. 2016;7(5):619–30.

[80] Thurfjell H, Ciuti S, Boyce MS. Applications of step-selection functions in ecology and conservation. Mov Ecol. 2014;2(1):4. 10.1186/2051-3933-2-4 PMC4267544 25520815

[81] Dougherty ER, Seidel DP, Carlson CJ, Spiegel O, Getz WM. Going through the motions: incorporating movement analyses into disease research. Ecol Lett. 2018;21(4):588–604. 10.1111/ele.12917 29446237

[82] Fagan WF, Krishnan A, Liao Q, Fleming CH, Liao D, Lamb C, Patterson B, Wheeldon T, Martinez-Garcia R, Menezes JF, Noonan MJ. Intraspecific encounters can lead to reduced range overlap. Mov Ecol. 2024;12(1):58. 10.1186/s40462-024-00501-w PMC11365178 39215311

[83] Wilber MQ, Yang A, Boughton R, Manlove KR, Miller RS, Pepin KM Wittemyer G. A model for leveraging animal movement to understand spatio‐temporal disease dynamics. Ecol Lett. 2022;25(5):1290–304. 10.1111/ele.13986 35257466

[84] Noonan MJ, Martinez-Garcia R, Davis GH, Crofoot MC, Kays R, Hirsch BT, Caillaud D, Payne E, Sih A, Sinn DL, Spiegel O. Estimating encounter location distributions from animal tracking data. Methods Ecol Evol. 2021;12(7):1158–73.

[85] Yang A, Wilber MQ, Manlove KR, Miller RS, Boughton R, Beasley J, Northrup J, VerCauteren KC, Wittemyer G, Pepin K. Deriving spatially explicit direct and indirect interaction networks from animal movement data. Ecol Evol. 2023;13(3):e9774. 10.1002/ece3.9774 PMC10040956 36993145

[86] Herraiz C, Triguero-Ocaña R, Laguna E, Jiménez‐Ruiz S, Peralbo‐Moreno A, Martínez‐López B, García‐Bocanegra I, Risalde MÁ, Vicente J, Acevedo P. Movement‐driven modelling reveals new patterns in disease transmission networks. J Anim Ecol. 2024;93(9):1275–87. 10.1111/1365-2656.14142 39004905

[87] Lane MR, Youngentob KN, Clark RG, Skewes JD, Marsh KJ. Home ranges and movements of an arboreal folivore after wildfire: comparing rehabilitated and non-rehabilitated animals in burnt and unburnt woodlands. Mov Ecol. 2024;12(1):75. 10.1186/s40462-024-00519-0 PMC11613491 39627876

[88] Melzer A, Carrick F, Menkhorst P, Lunney D, John BS. Overview, critical assessment, and conservation implications of koala distribution and abundance. Conserv Biol. 2000;14(3):619–28.

[89] Polkinghorne A, Hanger J, Timms P. Recent advances in understanding the biology, epidemiology and control of chlamydial infections in koalas. Vet Microbiol. 2013;165(3–4):214–23. 10.1016/j.vetmic.2013.02.026 23523170

[90] Quigley BL, Timms P. Helping koalas battle disease–Recent advances in Chlamydia and koala retrovirus (KoRV) disease understanding and treatment in koalas. FEMS Microbiol Rev. 2020;44(5):583–605. 10.1093/femsre/fuaa024 PMC8600735 32556174

[91] Russell I, Timms P, Hanger J, Loader J, Gillett A, Waugh C. Prevalence of Chlamydia pecorum in juvenile koalas (Phascolarctos cinereus) and evidence for protection from infection via maternal immunization. J Wildl Dis. 2018;54(4):863–5. 10.7589/2017-07-183 29733767

[92] McCann R, Bracken AM, Christensen C, Fürtbauer I, King AJ. The relationship between GPS sampling interval and estimated daily travel distances in chacma baboons (Papio ursinus). Int J Primatol. 2021;42(4):589–99.

[93] Beranek CT, Southwell D, Jessop TS, Hope B, Gama VF, Gallahar N, Webb E, Law B, McIlwee A, Wood J, Roff A. Comparing the cost-effectiveness of drones, camera trapping and passive acoustic recorders in detecting changes in koala occupancy. Ecol Evol. 2024;14(7):e11659. 10.1002/ece3.11659 PMC11219196 38957698

[94] Gillespie GR, Gallahar N, McIlwee AP, Hope BD, Webb EB, Clements M, Gama VF, Casley M, Jessop TS. Initial evaluation of a state-wide multi-method survey to estimate koala occupancy and abundance. Ecol Ind. 2025;176:113654.

[95] Howell LG, Clulow J, Jordan NR, Beranek CT, Ryan SA, Roff A, Witt RR. Drone thermal imaging technology provides a cost-effective tool for landscape-scale monitoring of a cryptic forest-dwelling species across all population densities. Wildl Res. 2021;49(1):66–78.

[96] Clough J, Prangell J, Fallon K, Beranek CT, Mikac KM. Abundance estimates of koalas (Phascolarctos cinereus) and southern greater gliders (Petauroides volans) on the Illawarra Range, New South Wales, from spatiotemporally-replicated thermal drone surveys. Australian Mammalogy. 2025;47(3):AM25026.

[97] Ryan SA, Southwell DM, Beranek CT, Clulow J, Jordan NR, Witt RR. Estimating the landscape-scale abundance of an arboreal folivore using thermal imaging drones and binomial N-mixture modelling. Biol Conserv. 2025;309:111207.

[98] Wagner B, Garnick SW, Ryan MF, Isaac JL, Begg A, Nitschke CR. Thermal drone surveys to detect arboreal fauna: Improving population estimates and threatened species monitoring. Ecol Appl. 2025;35(6):e70091. 10.1002/eap.70091 PMC12426585 40936356

[99] Duarte A, Adams MJ, Peterson JT. Fitting N-mixture models to count data with unmodeled heterogeneity: Bias, diagnostics, and alternative approaches. Ecol Model. 2018;374:51–9.

[100] Royle JA. N-mixture models for estimating population size from spatially replicated counts. Biometrics. 2004;60(1):108–15. 10.1111/j.0006-341X.2004.00142.x 15032780

[101] Chandler RB, Royle JA, King DI. Inference about density and temporary emigration in unmarked populations. Ecology. 2011;92(7):1429–35. 10.1890/10-2433.1 21870617

[102] O’Donnell KM, Thompson III FR, Semlitsch RD. Partitioning detectability components in populations subject to within-season temporary emigration using binomial mixture models. PLoS One. 2015;10(3):e0117216. 10.1371/journal.pone.0117216 PMC4361623 25775182

[103] Zhao Q, Royle JA. Dynamic N-mixture models with temporal variability in detection probability. Ecol Model. 2019;393:20–4.

[104] Neilson EW, Avgar T, Burton AC, Broadley K, Boutin S. Animal movement affects interpretation of occupancy models from camera-trap surveys of unmarked animals. Ecosphere. 2018;9(1):e02092.

[105] Kendall WL. Robustness of closed capture–recapture methods to violations of the closure assumption. Ecology. 1999;80(8):2517–25.

[106] Mezzini S, Fleming CH, Medici EP, Noonan MJ. How resource abundance and resource stochasticity affect organisms’ range sizes. Mov Ecol. 2025;13(1):20. 10.1186/s40462-025-00546-5 PMC11927164 40114205

[107] Santos Neto C, Dwyer RG, Michelot T, Cristescu RH. Effects of urbanisation on the movements of an arboreal specialist using hidden Markov models. Mov Ecol. 2025;13(1):66. 10.1186/s40462-025-00569-y PMC12487388 41029473

[108] Hradsky BA, Kelly LT, Robley A, Wintle BA. FoxNet: An individual-based model framework to support management of an invasive predator, the red fox. J Appl Ecol. 2019;56(6):1460–70.

[109] McLane AJ, Semeniuk C, McDermid GJ, Marceau DJ. The role of agent-based models in wildlife ecology and management. Ecol Model. 2011;222(8):1544–56.

