# Supplementary material for: Koalas in continuous time – unravelling the movement ecology of an iconic species using insights from a statewide telemetry program
Source: Mov Ecol. 2026 Aug 12;14:81. doi: 10.1186/s40462-026-00693-3 (PMC13746697; doi:10.1186/s40462-026-00693-3)
Supplement: Supplementary file 1 [file 40462_2026_693_MOESM1_ESM.docx]

*Table S1. The number of collars (Calibration Collars) and fixes (Fixes) used to estimate region-specific User Equivalent Range Errors (UEREs) for error calibration in fitted ctmm models. UERE values represent the root-mean-square location error (m) given ideal reception (i.e. PDOP = 1).*

| **Region** | **Calibration Collars** | **Fixes** | **UERE (95% CI)** |
| --- | --- | --- | --- |
| Port Macquarie | 12 | 3597 | 3.43 (3.38 – 3.49) |
| Narrandera | 20 | 9635 | 4.05 (4.01 – 4.09) |
| Richmond Range | 5 | 2016 | 3.23 (2.63 – 3.83) |
| Kanangra-Boyd | 18 | 5314 | 3.31 (3.26 – 3.35) |
| Georges River* | 13 | 2435 | 4.07 (4.00 – 4.13) |
| UNSCA* | 6 | 1204 | 4.07 (4.00 – 4.13) |
| Southern Tablelands | 23 | 8432 | 3.30(3.27 – 3.34) |
| **Total** | **97** | **32633** | **-** |

**The same error model was used for both Georges River and UNSCA due to their close proximity to each other relative to other regions.*

*Figure S1. Error calibration example for a female koala (SW-STL-038, Female, 6.5-7.5 years old, UNSCA region). Locations are coloured from old (blue) through to recent (red) and locations are represented as error ellipses relating to the predicted error after error calibration.*

*Figure S2. Example demonstrating the influence of clear extra-territorial forays on home range estimation. The non-breeding season home range estimated using the full dataset (including 5 distinct forays, orange, left) has a low effective sample size (DOF), wide confidence intervals and an ecologically implausible point estimate an order of magnitude larger than conspecifics in the same region. In contrast, despite having a smaller nominal sample size, the non-breeding season home range size estimated using the dataset with forays removed (green, right) has a four-fold higher effective sample size, greater precision and an ecologically plausible home range size.*

*Table S2. The total number of locations before and after removal of extra-territorial forays per region alongside the proportional reduction in total fixes removed during filtering.*

| **Region** | **Pre-filtering** | **Post-filtering** | **Proportion removed (%)** |
| --- | --- | --- | --- |
| Port Macquarie | 56770 | 51844 | 8.7 |
| Narrandera | 27327 | 26242 | 4.0 |
| Richmond Range | 75426 | 75163 | 0.3 |
| Kanangra-Boyd | 31833 | 29945 | 5.9 |
| Georges River | 37019 | 36241 | 2.1 |
| UNSCA | 39079 | 36425 | 6.8 |
| Southern Tablelands | 49081 | 47094 | 4.0 |
| **Total** | **316535** | **302945** | **4.3** |

*Figure S3. Example of variograms fit to a range resident (top) and transient (bottom) koala. Note the continued increase in semivariance at increasing time lags in the transient animal, indicating that this animal does not display typical range residency behaviour.*

| **Region** | **Sex** | **OU** | **OUF** |
| --- | --- | --- | --- |
| Port Macquarie | Male | 1 | 7 |
| Port Macquarie | Female | 6 | 11 |
| Narrandera | Male | 8 | 10 |
| Narrandera | Female | 7 | 24 |
| Richmond Range | Male | 0 | 6 |
| Richmond Range | Female | 2 | 8 |
| Kanangra-Boyd | Male | 5 | 6 |
| Kanangra-Boyd | Female | 12 | 10 |
| Georges River | Male | 0 | 6 |
| Georges River | Female | 6 | 11 |
| UNSCA | Male | 3 | 6 |
| UNSCA | Female | 0 | 11 |
| Southern Tablelands | Male | 2 | 13 |
| Southern Tablelands | Female | 1 | 30 |
| **Total** | **Male** | **19** | **54** |
|  | **Female** | **34** | **105** |

*Table S3. Supported movement models of range-resident koalas stratified by region and sex. OU = Ornstein-Uhlenbeck movement process that features autocorrelation in an animal’s position but no autocorrelation in velocity; OUF = Ornstein-Uhlenbeck Foraging movement process that features autocorrelation in both position and velocity.*

*Table S4. Minimum, maximum and mean effective sample sizes for diffusion rate and home range estimation across study regions.*

| **Region** | **Diffusion Rate** | | | **Home Range** | | |
| --- | --- | --- | --- | --- | --- | --- |
|  | **Min** | **Max** | **Mean** | **Min** | **Max** | **Mean** |
| Port Macquarie | 25 | 726 | 294 | 3 | 44 | 16 |
| Narrandera | 15 | 1097 | 215 | 5 | 347 | 43 |
| Richmond Range | 48 | 1159 | 364 | 4 | 247 | 33 |
| Kanangra-Boyd | 67 | 1486 | 533 | 12 | 193 | 54 |
| Georges River | 66 | 1772 | 545 | 5 | 89 | 34 |
| UNSCA | 61 | 1203 | 473 | 5 | 68 | 24 |
| Southern Tablelands | 28 | 1493 | 462 | 7 | 101 | 38 |

*Table S5. Population mean coefficient of variation (CoV) for estimates of home range size (Range), diffusion rate (D), range crossing time (Ƭ_p_) and directional persistence (Ƭ_v_) by sex (M = male, F = female) and region. Values in parentheses represent 95% confidence intervals.*

| **Region** | **Sex** | **Range** | **D** | **Ƭ_p_** | **Ƭ_v_** |
| --- | --- | --- | --- | --- | --- |
| Port Macquarie | M | 0.73 (0.25-1.21) | 0.94 (0.41-1.48) | - | 1.20 (0.36-2.07) |
| Port Macquarie | F | 0.72 (0.41-1.04) | 0.70 (0.44-0.95) | 0.48 (0.24-0.72) | 0.66 (0.29-1.03) |
| Narrandera | M | 0.64 (0.39-0.89) | 0.73 (0.46-1.00) | 0.72 (0.44-1.01) | 0.64 (0.22-1.07) |
| Narrandera | F | 1.03 (0.66-1.38) | 1.05 (0.71-1.38) | 0.36 (0.20-0.52) | 0.73 (0.44-1.02) |
| Richmond Range | M | - | 0.51 (0.21-0.82) | 0.55 (0.14-0.97) | - |
| Richmond Range | F | 0.89 (0.41-1.39) | 0.53 (0.28-0.77) | 0.48 (0.21-0.76) | 0.57 (0.22-0.93) |
| Kanangra Boyd | M | 0.44 (0.23-0.66) | 0.82 (0.43-1.20) | 0.59 (0.31-0.86) | - |
| Kanangra Boyd | F | 0.89 (0.56-1.22) | 0.82 (0.54-1.10) | 0.43 (0.27-0.59) | 0.56 (0.24-0.88) |
| Georges River | M | 0.56 (0.16-0.96) | 0.99 (0.35-1.66) | 0.78 (0.27-1.30) | 0.39 (0.12–0.67) |
| Georges River | F | 0.50 (0.29-0.70) | 0.41 (0.27-0.55) | 0.53 (0.31-0.75) | 1.09 (0.5-1.68) |
| UNSCA | M | 0.54 (0.25-0.84) | 0.73 (0.36-1.09) | 0.56 (0.23-0.89) | 0.82 (0.26-1.40) |
| UNSCA | F | 0.84 (0.38-1.32) | 0.72 (0.39-1.05) | 0.35 (0.15-0.56) | 0.87 (0.43-1.31) |
| Southern Tablelands | M | 0.68 (0.40-0.96) | 0.58 (0.35-0.80) | 0.54 (0.30-0.77) | 0.63 (0.33-0.93) |
| Southern Tablelands | F | 0.59 (0.42-0.77) | 0.48 (0.35-0.61) | 0.49 (0.33-0.64) | 0.71 (0.47-0.95) |

**CoV was unable to be estimated in some cases due to limited sample sizes.*
